# Chemical Composition of *Cinnamomum verum* Leaf and Flower Essential Oils and Analysis of Their Antibacterial, Insecticidal, and Larvicidal Properties

**DOI:** 10.3390/molecules26206303

**Published:** 2021-10-19

**Authors:** Arunaksharan Narayanankutty, Krishnaprasad Kunnath, Ahmed Alfarhan, Rajakrishnan Rajagopal, Varsha Ramesh

**Affiliations:** 1 Division of Cell and Molecular Biology, PG and Research Department of Zoology, St. Joseph’s College (Autonomous), Devagiri, Calicut 673008, India; 2 Department of Pharmacology, Devaki Amma Memorial College of Pharmacy, Chelembra, Malapuram 673634, India; Krishnaprasadk91@gmail.com; 3 Department of Botany and Microbiology, College of Science, King Saud University, P.O. Box 2455, Riyadh 11451, Saudi Arabia; rrajagopal@ksu.edu.sa; 4 Department of Biotechnology, Deakin University, Geelong, VIC 3217, Australia; vramesh@deakin.edu.au

**Keywords:** *Cinnamomum verum*, leaf essential oil, flower essential oil, fumigant activity, repellent effect, antibacterial activity, larvicidal activity

## Abstract

*Cinnamomum verum* is widely used in traditional medicines, and the different parts of the plant, such as bark, leaves, and flowers, are used for essential oil production. The present study compared the chemical composition of the essential oil of *C. verum* extracted from the leaves and flowers. In addition, efficacy of these essential oils against the two common pests *Sitophilus oryzae* and *Callosobruchus maculatus* was also evaluated. The results indicated the presence of cinnamaldehyde, eugenol, caryophyllene, and linalool in these essential oils, however, at different concentrations. The leaf essential oil was found to be 10–20% more effective as a fumigant against both the pests. Likewise, the leaf essential oil found to repel these pests even at lower concentrations than that of flower essential oil of *C. verum*. Besides, these essential oils were also effective in controlling the growth of various gram positive and gram negative microbial pathogens and possibly a safeguard for human health. On contrary, both the essential oils were found to be safe for the application on grains, as indicated by their germination potentials. It was also observed that these essential oils do not cause any significant toxicity to guppy fishes, thus confirming their ecological safety for use as a biopesticide.

## 1. Introduction

Essential oils are important pharmacological and commercial agents that are widely utilized for various industrial purposes, cosmetic uses, and medicinal applications, including aromatherapy [1]. Various essential oils are utilized as ecofriendly agents against varying classes of pests, vectors, and other insect agents [2]. Aromatic plants, especially spices, are the primary sources of essential oil with plenty of biological activities. Essential oils of Cinnamomum plants are widely used as promising antibacterial agents. Reports have indicated that the essential oils of *C. camphora* [3] and *C. cassia* [4,5] reverse antibiotic resistance and biofilm formation. Studies have also indicated that the *C. zeylanicum* (synonym of *C. verum*) has also found to be effective against multidrug resistant strains of clinical Shigella isolates [6]. Likewise, the *C. cassia* essential oil in combination with polymyxin B effectively reduces the growth of *Klebsiella pneumoniae* and *Serratia marcescens* [7]. Studies by Yap, Krishnan, Chan, and Lim [8] have indicated the antibacterial activity against drug resistant E. coli strains. Antifungal potentials were also attributed to the essential oils of *C. zeylanicum* [9] and *C. cassia* [10,11].

Pests and vectors are major threats to the global food and health security, the risk of which is higher in developing countries compared to the developed nations [12,13]. Insect pests are major organisms with significant impact on the economy and agricultural productivity of a country [14]. These insect pests are known to inhibit the plant growth, grain production, and damage the stored products; the common insect pests of stored food products accounting for the severe damages include *Sitophilus oryzae* and *Callosobruchus maculatus* [15]. The attack of pests in conditions, such as pest outbreaks, can lead to the reduced production of food crops and lead to shortage of food sources. In addition, the damage of pest attack is more prominent in the case of stored products as it can damage the already produced materials. The majority of the global food supply is in the form of grains and cereals, as well as pulses [16]. There are several chemical pesticides are being used for the control of various pests: the pyrethroids, phosphines, and organochlorines [17,18]. These pesticides are known to cause several toxic effects to neural and other organ systems [19,20,21].

The vectors are other important organisms, primarily insects that are known to spread the diseases including dengue, chikungunya, malaria, plague, and so many others [22]. Among these, the mosquitoes are considered to be the most potent, with a higher propensity of mortality associated with vector borne diseases. Further, there are numerous diseases and pathogenic organisms that are passing from one person to another via these vector organisms. Among these, the mosquito vectors, such as *Aedis*, *Anopheles*, and *Culex*, are considered to be the most important. However, the roles of mosquito species, such as *Armigeres* sp, are emerging risk factors over the recent years.

Apart from the general bacterial, viral, and fungal diseases that are spreading through vectors, there are several other microbial diseases that are associated with elevated mortality [23]. In addition, the evolution of antibiotic insensitive microbes enhances the threat to present day health care systems. Despite the introduction of various antibiotics, microbial pathogens have gained the resistance against these antimicrobial compounds [24,25]. Several microbial pathogens are reported to be associated with human diseases; among these, some of the most relevant are *E. coli*, *Staphylococcus aureus*, *Pseudomonas sp.*, etc.

The study, therefore, analyzed the antibacterial activity and possible use of biologically safe pesticide and larvicide agent. The insecticidal potential was analyzed against different pests of stored products, such as *Sitophilus oryzae* and *Callosobruchus maculatus*. The biological safety was evaluated in non-targeted organisms and also using grain germination potential.

## 2. Results

### 2.1. Percentage Yield of Essential Oil and Chemical Composition

The yield of leaf (LEO) and flower (FEO) essential oil by steam distillation was 1.44 ± 0.09 and 1.07 ± 0.12%. Both the essential oils possessed similar volatile components, with a different percentage composition. The common compounds among these two essential oils were the cinnamaldehyde, eugenol, and linalool (Table 1). The most abundant (< 10%) compounds in the LEO were (*E*) Cinnamaldehyde (35.6%), linalool (18.92%), eugenol (18.69%), and (*E*) Cinnamyl acetate (12.5%). In the FEO, compounds with highest abundance were (*E*) Cinnamaldehyde (42.88%), eugenol (21.33%), and linalool (15.62%).

### 2.2. Anti-Microbial Screening of the Leaf and Flower Essential Oils of C. verum

Antibacterial properties of the *C. verum* essential oils were evaluated against *E. coli*, *S. aureus*, *P. aeruginosa*, and *Salmonella enteritidis* (Table 2). The leaf essential oil was found to have higher zone of inhibition compared to the *Cinnamomum verum* leaf essential oil (FEO), except against salmonella strain. Similarly, as indicated in the Table 2, the *Cinnamomum verum* leaf essential oil (LEO) was found to be more effective and had the lowest minimum inhibition concentration (MIC) values compared to the *Cinnamomum verum* leaf essential oil (FEO) (*p* < 0.05).

### 2.3. Efficacy of C. verum Essential Oils as Possible Larvicidal Agent

The three mosquito species (*Armigeres*, *Aedes*, and *Culex*) were chosen for the larvicidal screening of the LEO and FEO (Table 3). Among all the three mosquito species, the LEO was found to be more effective compared to the flower essential oil of *C. verum*.

In Armigeres species, the IC_50_ values of LEO (88.34 ± 2.9 µg/mL) was significantly higher (*p* < 0.01) than the FEO (97.71 ± 3.6 µg/mL). Similarly, the activity of LEO as expressed in terms of IC_50_ values against Aedes (41.56 ± 4.1 µg/mL) and Culex (64.88 ± 1.9 µg/mL) species were significantly higher than the FEO.

### 2.4. Insecticide Properties of the Leaf and Flower Essential Oils of C. verum

**Figure 1:**
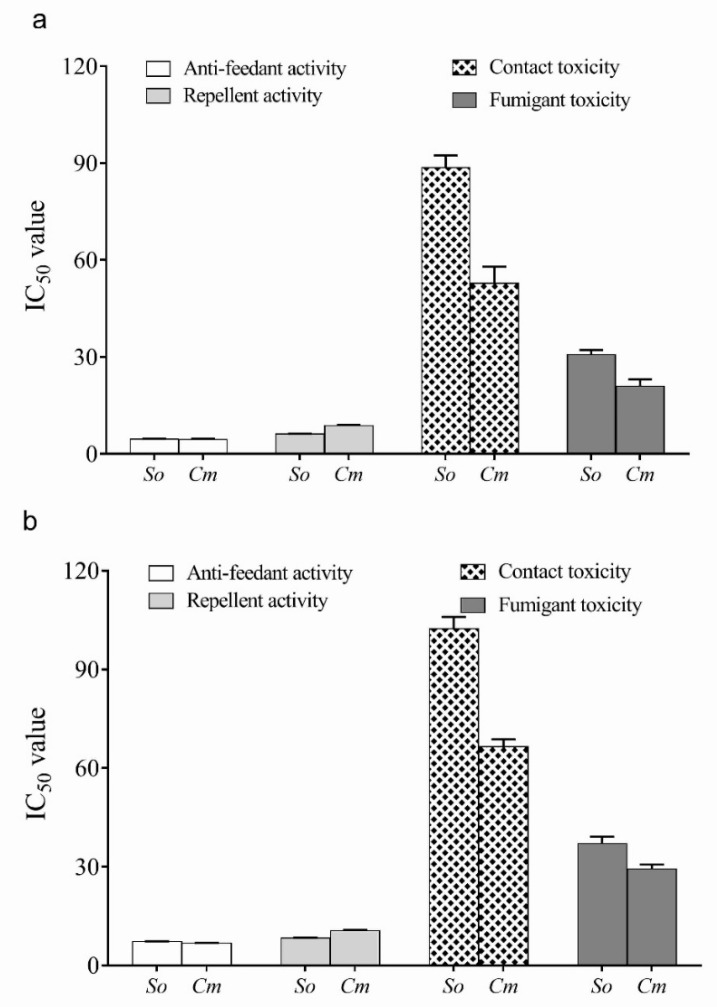
Insect repellent and insecticidal properties of the *Cinnamomum verum* flower (FEO) (**a**) and leaf (LEO) (**b**) essential oils against the pests of stored grains *Sitophilus oryzae* (So) and *Callosobruchus maculatus* (Cm). The values shown on the Y-axis is the IC_50_ or half-maximal inhibition concentrations for respective biological activities compared to the untreated control group.

The rice weevil *Sitophilus oryzae* was found to be more resistant compared to the *C. maculatus* from the toxicities of various essential oils (Figure 1). The application of flower essential oil of *C. verum* was more effective as an anti-feedant and repellant agent against *S. oryzae* (4.58 ± 0.15 and 6.19 ± 0.07 µg/L of air). Similarly, the fumigant and contact toxicity was also high in the FEO (88.64 ± 3.73 and 30.72 ± 1.45 µg/L).

A similar trend was also observed against *C. maculatus*; the half-maximum inhibition concentration for antifeedant activity, repellency, fumigant, and contact toxicity were found to be 4.52 ± 0.17, 8.80 ± 0.18, 52.81 ± 5.11, and 20.97 ± 2.05 µg/L, respectively.

### 2.5. Phytotoxicity of Essential Oil on Germinating Seeds

The leaf and flower essential oils of *C. verum* were found to be highly active against the mosquito larvae and various other pests. To elucidate the possible phytotoxic properties of the essential oils, the impact of wheat germination potential was evaluated; however, till 144 h, there observed no significant alterations in the percentage viability of grains in control and different essential oil treated groups (Table 4).

## 3. Discussion

Pests, vectors, and parasites are economically important in the areas of health care and agriculture/food sciences [26]. Both these organisms are predominated by different insects; hence, control of the insect pests/vectors is essential for the sustainable development of a country in terms of agricultural development, food security, and improvements in the health care status of people. Chemical pesticides are often reported to be phytotoxic to the vegetation [27] and animals, as well as humans [19,21]. The recent reports highlight the application of natural products, especially the volatile compounds for the repellency/control of these insects from the settlement areas [28]. Essential oils are the compounds with high volatility, as well as good aroma; their use is often encouraged as an insect repellant and insecticidal agent [2]. Cinnamomum is a genus of plants that is well-elucidated for their essential oils producing abilities and their applications in the control of various insect pests/vectors. However, the *C. verum* essential oils are less explored for such activities using experimental studies.

The present study indicated a higher potential of *C. verum* flower and leaf essential oils as potential insect repellents, especially against the pests of stored grains. The application of the essential oils was also capable of inhibiting the feed intake by these pests. *Cinnamomum zeylanicum* has already been shown to have repellent activity against *Diaphania hyalinata* [29]. Previous studies have also indicated similar repellant activity for *C. camphora* essential oil [30]; besides, the insecticidal property was also exhibited by the essential oil against *S. oryzae* and *Bruchus rugimanus*. Further, the results of the study also indicated the fumigant and contact toxicity of both the essential oils of *C. verum* against the selected stored pests, with a higher efficacy by the FEO. Previous studies by Jeon, Lee, and Lee [31] have indicated the possible insecticidal potential of the bark essential oil of *c. zeylanicum* against *Dermatophagoides*, *Tyrophagus*, and *Ricania*. Similar insecticidal property has been also shown by *C. cassia* against the insect vectors, such as mosquitoes [32]. Likewise our results also observed significant larvicidal potential of the *C. verum* essential oils; unlike their insecticidal property, the higher activities were observed for the LEO compared to the FEO. Previously, the bark essential oils of *C. cassia*, *C. verum*, *C. loureiroi* [33], and *C. osmophloeum* [34,35] were observed against the larvae of different species of mosquitoes. It is, thus, possible that the larvicidal properties of these essential oils may be enhancing the value of *C. verum* essential oils as a mosquito control agent.

Further, the essential oils extracted from the leaves and flowers of the plant were capable of inhibiting the microbial growth in the various bacterial strains including that of *E. coli*, *Salmonella*, *Staphylococcus*, and *Pesudomonas*; the leaf essential oil was more potent antibacterial agent over FEO. Reports have also indicated the antimicrobial properties of *C. verum* (or *C. zeylanicum*) had been previously reported in the multi-drug resistant strains of Shigella [6], *Candida* sp. [9], and other bacterial strains [36,37]. Bark essential oil of *C. verum* was found to destroy the membrane of *Klebsiella* sp by inducing oxidative damages [38]. It is, therefore, likely that, apart from the bark essential oils, the leaf and flower essential oils are also strong antimicrobial agents against different gram positive and gram negative bacteria.

Results also indicated the presence of bioactive compounds, such as eugenol, cinnamaldehyde, and linalool, in flower and leaf essential oils; the leaf essential oil is found to have higher proportion of linalool in LEO and eugenol in FEO. Previous studies have indicated that *Cinnamomum zeylanicum* (a synonym of *C. verum*) is rich in cinnamaldehyde and its derivatives, followed by linalool, caryophyllene, and eugenol [39]. It is, therefore, possible that the composition variation between the leaf and flower essential oils of *C. verum* may be responsible for its differential activities observed.

Overall, the bioactivities of both leaf and flower essential oils of *C. verum* are promising as insecticidal, larvicidal, and antimicrobial agent; besides, the non-toxic nature of these oils are also observed in germinating grains. Hence, it can be presumed that the *C. verum*-derived essential oil may act as a source of value added and environmentally safe products in pest and vector control in the coming years.

## 4. Materials and Methods

### 4.1. Plant Materials Collection, Essential Oil Extraction, and Phytochemical Analysis by GC-MS

*Cinnamomum verum* leaves and flowers were collected from the Kerala Agriculture University, Thrissur, Kerala. The collected leaves and flowers were the washed, frozen, and powdered under −80 °C. About 500 g of the leaves and 100 g buds were weighed in a digital balance and then extracted by steam distillation methods that are previously mentioned in the article of Nagarajan et al. [40] for 5 h. The yield of essential oil was expressed in percentage (mL of essential oil yielded from 100 g plant material). The GC-MS analysis was carried out according to the conditions mentioned in the previous article by Nagarajan et al. using a SPB-1 column [40]. The percentage composition was estimated from the relative peak area obtained from the GC-MS chromatogram.

### 4.2. Antibacterial Activity of the C. verum Leaf and Flower Essential Oil

The flower and leaf essential oils were evaluated for their antimicrobial activity against different strains of bacteria and fungi. The antibacterial activity was done as agar disc-diffusion method [41] and minimum inhibitory concentration [42] as described previously. The experiment was conducted in triplicate and repeated for five times to ensure accuracy. The microbial strains used in the study were relevant to the human diseases, which include *Escherichia coli*, *Staphylococcus aureus*, *Pseudomonas aeruginosa*, and *Salmonella enteritidis*.

### 4.3. Larvicidal Potential of Essential Oils from the Leaves and Flowers of C. verum

Different mosquito cultures of *Culex tritaeniorhynchus*, *Armigeres subalbatus*, and *Aedes aegypti* were collected and maintained under normal atmospheric conditions. The larval stages of each of the mosquito were maintained in glass jars and those in the third instar stage of development were used for the larvicidal studies. About 50 larvae from each culture were transferred to individual chambers, and different concentrations (0–100 µg/mL) of essential oils were transferred to these glass chambers and maintained for 24 h. The average mortality in each concentration was counted and LC_50_ value was estimated. The experiment was repeated three times each conducted in triplicate.

### 4.4. Insecticidal Potential of the Leaf and Flower Essential Oil of C. verum

The pests of stored grains were chosen as the model organisms; they include common rice weevil (*Sitophilus oryzae*) and pulse beetle (*Callosobruchus maculatus*). Anti-feedant potential of the different essential oils was carried out as per the protocols of Yan et al. [43]. The fumigant effect of leaf and flower essential oils of *C. verum* was evaluated using the standard protocols previously described in the article of Bhavya et al. [44]. The repellant activity of leaf and flower essential of *C. verum* was determined according the protocols described by Kłyś et al. [45]. Contact toxicity profiles of the different essential oils were carried out by the methods of Patiño-Bayona et al. [46]. The half-maximal inhibition concentration of LC50 value was calculated using Probit analysis.

The IC_50_ values were expressed in terms of μg/g of food (anti-feedant activity), μg/L of air (fumigant toxicity and repellent activity), μg/mm^2^ area (contact toxicity).

### 4.5. Phytotoxic Potential of the Leaf and Flower Essential Oil of C. verum

The effect of leaf and flower essential oil of *C. verum* on the germination potential of wheat grains was evaluated for 144 h [30]. Briefly, the wheat grains planted in individual plates were exposed to 200 and 500 µg/mL of these essential oils, and the percentage of germination at each tested doses was determined by comparing with that of a control.

### 4.6. Statistical Analysis

The extraction of individual essential oils was carried out by steam distillation in three individual sets of process. The results were represented as Mean± SD, and, using Student’s *t*-test, the statistical analysis of the data was performed.

## 5. Conclusions

In conclusion, the results are supportive to the fact that the leaf and flower essential oils of *C. verum* are rich sources of various chemical volatiles, including Cinnamaldehyde. Further, these molecules are found to have strong insecticidal, larvicidal, and antimicrobial properties. It is also noteworthy that these essential oils have no toxic effects observed and are also found to be safe in germinating grains. Hence, it is possible that the *C. verum* essential oils may evolve as a promising green pesticide and antimicrobial agent in the near future.

## Figures and Tables

**Table 1:** GC-MS analysis of *Cinnamomum verum* LEO and FEO essential oils.

No.	Compound	Kovats Index (KI)	% ^a^	
			LEO ^b^	FEO ^c^
1	Camphene	945	0.24	0.41
2	*β*-pinene	967	0.33	0.14
3	Sabinene	972	0.85	0.22
4	Myrcene	988	1.54	1.99
5	1,4-Cineole	1010	0.52	0.13
6	Limonene	1020	0.38	0.64
7	*Cis-β*-Ocimene	1024	0.08	0.11
8	*trans-β*-Ocimene	1028	0.07	0.15
9	*p*-Cymene	1018	1.88	2.68
10	Linalool	1095	18.92	15.62
11	*γ*-Terpinene	1054	0.19	0.55
12	*α*-Terpineol	1296	0.84	1.33
13	Piperitone	1247	0.22	0.41
14	Geraniol	1254	0.62	0.18
15	(*E*)-Cinnamaldehyde	1262	35.6	42.88
16	(*Z*)-Cinnamaldehyde	1271	0.65	0.88
17	Eugenol	1358	18.69	21.33
18	(*E*)-Cinnamyl acetate	1443	12.5	8.26
19	Eugenyl acetate	1496	1.38	0.74
20	Benzyl benzoate	1754	0.25	0.22

^a^ Relative area = relative contents expressed as percentages of the total oil composition. ^b^ LEO—essential oil obtained from leaves. ^c^ FEO—essential oil obtained from flowers.

**Table 2:** Antibacterial activity of *C. verum* leaf (LEO) and flower (FEO) essential oils in terms of zone of inhibition and the Minimum inhibitory concentrations (μg/mL).

Bacteria	Zone of Inhibition (mm)		MIC (μg/mL)	
	LEO	FEO	LEO	FEO
*Escherichia coli*	14.9 ± 0.1 *	11.2 ± 0.3	2.7 ± 0.1 *	3.2 ± 0.2
*Staphylococcus aureus*	17.2 ± 0.3 *	15.2 ± 0.5	2.8 ± 0.4 *	2.9 ± 0.2
*Pseudomonas aeruginosa*	17.6 ± 0.4 *	16.4 ± 0.2	3.3 ± 0.2 *	4.1 ± 0.3
*Salmonella enteritidis*	19.2 ± 0.3	18.9 ± 0.1	3.7 ± 0.2 *	4.0 ± 0.2

(* indicate significance variation *p* < 0.05).

**Table 3:** Mosquito larvicidal activity of *C. verum* leaf (LEO) and flower (FEO) essential oils and expressed as LC_50_ value (μg/mL).

Mosquito	LC_50_ (μg/mL)	
	LEO	FEO
*Armigeres subalbatus*	88.34 ± 2.9 **	97.71 ± 3.6
*Aedes aegypti*	41.56 ± 4.1 **	58.04 ± 2.8
*Culex tritaeniorhynchus*	64.88 ± 1.9 ***	80.66 ± 3.6

(** indicate *p* < 0.01; *** indicate *p* < 0.001).

**Table 4:** The toxicity analysis of flower (FEO) and leaf (LEO) essential oil of *Cinnamomum verum* in the germinating wheat grains (estimated as germination index) (ns- indicate no significant variation with the negative control).

Duration of Exposure in Hours	Negative Control	*Cinnamomum verum* Essential Oil (µg/mL)			
		LEO		FEO	
		250	500	250	500
48	11.4 ± 1.2	10.9 ± 1.1^ns^	10.0 ± 1.5 ^ns^	12.2 ± 2.1 ^ns^	14.3 ± 2.2 ^ns^
72	27.7 ± 2.3	27.8 ± 2.4 ^ns^	25.3 ± 2.0 ^ns^	26.8 ± 2.0 ^ns^	30.4 ± 2.0 ^ns^
96	59.4 ± 1.4	59.3 ± 1.8 ^ns^	57.3 ± 0.8 ^ns^	55.4 ± 0.8 ^ns^	62.1 ± 3.4 ^ns^
120	78.8 ± 2.1	79.2 ± 1.5 ^ns^	78.8 ± 0.9 ^ns^	74.3 ± 2.4 ^ns^	72.4 ± 3.1 ^ns^
144	88.9 ± 2.3	86.9 ± 1.3 ^ns^	85.9 ± 2.1 ^ns^	84.3 ± 3.2 ^ns^	86.2 ± 2.7 ^ns^

## Data Availability

The data may be shared upon valid request.

## Sample Availability

Samples are available from A.N. on valid request.
